# Workplace Social Capital Subscales and Their Association With Depressive Symptoms in Japanese Research Institutions

**DOI:** 10.7759/cureus.85945

**Published:** 2025-06-13

**Authors:** Asako Matsuura, Shin-ichiro Sasahara, Shotaro Doki, Daisuke Hori, Yu Ikeda, Norishige Kanai, Wakako Migaki, Hotaka Tsukada, Soshi Takao

**Affiliations:** 1 Graduate School of Comprehensive Human Sciences, University of Tsukuba, Tsukuba, JPN; 2 Division of Translational Nursing, Toho University, Funabashi, JPN; 3 Occupational and Aerospace Psychiatry Group, University of Tsukuba, Tsukuba, JPN; 4 Public Health, Okayama University, Okayama, JPN

**Keywords:** bonding social capital, bridging social capital, depressive symptoms, linking social capital, mental health, tsukuba salutogenic occupational cohort study (t-socs), workers, workplace social capital

## Abstract

Background

The challenges to workers' mental health in rapidly changing modern work environments have highlighted the crucial role of workplace social capital (WSC). However, a comprehensive examination of how its structural dimensions (bonding, bridging, linking) relate to depressive symptoms, particularly among Japanese workers and within specific occupational settings such as research institutions, is insufficient. While previous studies have explored WSC, few have rigorously differentiated these three dimensions and their independent associations with mental health outcomes, especially in the Japanese context, where unique work culture dynamics may play a role.

Objective

This study aimed to cross-sectionally evaluate the association between each structural dimension of WSC (bonding, bridging, linking) and depressive symptoms among workers in research institutions and related organizations in Japan.

Methods

A cross-sectional web-based survey (Tsukuba Salutogenic Occupational Cohort Study: T-SOCS) was conducted in 2022, targeting 21,875 workers in institutions located in Tsukuba Science City. A total of 3861 responses were received (response rate: 17.7%), and data from 3145 valid responses with complete data were analyzed. WSC was assessed using the Japanese version of an eight-item scale to calculate the bonding, bridging, and linking scores. Depressive symptoms were evaluated using the Japanese version of the Patient Health Questionnaire-9 (PHQ-9), with a score ≥ 5 defined as “having depressive symptoms.” Multiple logistic regression analysis was performed to examine the association between each WSC dimension and depressive symptoms, adjusting for personal attributes (biological sex, age, marital status, and caregiving/child presence) and occupational characteristics (employment type, job type, managerial position, and flextime availability).

Results

Of the participants, 47.2% were identified as having depressive symptoms (PHQ-9 ≥ 5). After adjustment for personal and occupational factors, higher bonding WSC scores were significantly associated with lower odds of having depressive symptoms (odds ratio (OR) = 0.89; 95% CI: 0.81-0.97; p = 0.01). Similarly, higher bridging WSC scores were significantly associated with lower odds of having depressive symptoms (OR = 0.90; 95% CI: 0.81-0.99; p = 0.03). In contrast, linking WSC score showed no significant association with depressive symptoms (OR = 0.95; 95% CI: 0.89-1.01; p = 0.11). Being female, providing care, and working in administrative roles were also associated with increased odds of depressive symptoms.

Conclusion

In this sample of Japanese research institution workers, higher levels of horizontal WSC (bonding and bridging) were associated with fewer depressive symptoms, whereas vertical WSC (linking) was not. These findings underscore the importance of fostering horizontal SC for workers' mental health. However, the study's cross-sectional design, low response rate, and specific population limit the generalizability of our findings and preclude causal inference. Future research should employ longitudinal designs to establish causality, expand to diverse occupational groups, and explore the cultural nuances and potential mediating factors that could inform more targeted interventions.

## Introduction

In contemporary society, the work environment is rapidly changing owing to globalization and technological innovation, demanding high adaptability from workers [1]. In particular, the spread of digital technology, while increasing flexibility in work styles, blurs the boundaries between work and private life, potentially strengthening the association between the work environment and mental health [2]. Workplace stress and interpersonal relationships are major factors associated with workers' mental health [3-5].

Social capital (SC) is defined as "social connections and the norms and trust that arise from them" [6], and positive associations with health have been suggested [7]. Workplace social capital (WSC) represents the quality and structure of social relationships within an organization [7].

In recent years, WSC has garnered attention as a workplace environmental factor. WSC refers to intangible resources within the workplace, such as trust, norms of reciprocity, and networks, and has been reported to be associated with a reduced likelihood of mental health problems [8,9]. Previous studies have reported that low WSC is associated with an increased likelihood of developing depression [10].

Workers' mental health problems not only diminish individual quality of life but also lead to significant economic losses due to work absence and reduced productivity [11-15], highlighting the importance of primary prevention for mental health maintenance and promotion [16,17]. Mental health is complexly linked to personal factors [18,19], workplace factors [20-22], and family factors [23,24].

SC is defined as "social connections and the norms and trust that arise from them" [23], and positive associations with health have been suggested [24]. WSC represents the quality and structure of social relationships within an organization [24].

WSC was traditionally conceptualized through structural (structure and density of networks), relational (trust, norms of reciprocity), and cognitive (shared goals and values) dimensions [25]. However, the existence of different dimensions has recently been proposed: bonding (relationships among colleagues), bridging (relationships with different departments or external collaborators), and linking (relationships between workers and managers) [26]. This latter framework is argued to offer a more nuanced understanding of workplace interpersonal dynamics and their specific impacts.

Bonding SC concerns horizontal relationships among colleagues, such as within departments or peer groups. It can enhance team cohesion and cooperation for smoother daily tasks and provide emotional support and empathy, potentially reducing loneliness. Conversely, excessive bonding may foster exclusivity or peer pressure, causing stress for some.

Bridging SC involves relationships across diverse groups, such as collaborators from different professional backgrounds or departments. This can enable access to varied information and resources, potentially improving innovation and problem-solving. Such connections may also positively impact mental health by offering diverse perspectives, thereby enhancing job control and self-efficacy.

Linking SC pertains to vertical relationships, such as between workers and managers. It can influence participation in organizational decision-making and resource allocation, contributing to employee empowerment and operational efficiency. While linking SC can enhance engagement and mental health through supervisor support and fair evaluations, it may also be negatively associated with mental health owing to issues such as harassment or unfair treatment from superiors [27].

Although WSC has been linked to various outcomes such as productivity and mental well-being [28-36], previous research has often not differentiated between these three crucial dimensions. Many studies, including those conducted in Japan, have either focused on WSC as a unidimensional construct or have not simultaneously examined the distinct contributions of bonding, bridging, and linking capital to mental health outcomes while controlling for a wide range of covariates [37,38]. This lack of differentiation has left it unclear how each dimension uniquely contributes to, or potentially undermines, employee mental health.

Herein lies a critical gap and the novelty of our study. A comprehensive investigation into their individual associations is particularly vital within the Japanese context, where workplace culture norms (e.g., hierarchical structures, emphasis on group harmony) may create a unique interplay among these dimensions. For instance, a culture valuing harmony might foster strong bonding capital but inadvertently stifle the bridging or linking capital necessary for accessing new information or resources, with unknown net effects on mental health. This study addresses this gap by being one of the first to disentangle these three dimensions of WSC in a sample of workers from Japanese research institutions, a setting with its own distinct organizational features, where collaboration (bridging) and access to leadership (linking) are arguably as critical as collegial support (bonding).

The objective of this study was to cross-sectionally assess the association between each dimension of WSC (bonding, bridging, and linking) and depressive symptoms among workers employed in research institutions and related organizations. The study aimed to provide initial insights that could inform the development of more targeted and effective workplace mental health strategies focusing on the distinct roles of different WSC dimensions.

## Materials and methods

Participants

This study utilized cross-sectional data from the baseline survey of the Tsukuba Salutogenic Occupational Cohort Study (T-SOCS). The survey was conducted as part of an initiative by the Occupational Health Committee of the Tsukuba Science City Network Council, which aims to promote mental health among workers in the region.

Between January and March 2022, we targeted 21,875 workers from institutions, including research institutions, educational institutions, private companies, and local governments, affiliated with the Council that had consented to participate. The recruitment was conducted as follows: a designated representative from each participating institution distributed an email to their employees. This email contained a detailed explanation of the study's purpose and procedures, assurance of anonymity, and a direct link to the web-based questionnaire. Participants accessed the survey via the link, read the information sheet on the first page, and provided their electronic consent before proceeding to the questionnaire. Only responses with complete data for the variables used in this analysis were included.

Survey content

The web-based questionnaire collected information on the following items: basic attributes of biological sex, age, and marital status; presence/absence of caregiving responsibilities; presence/absence of preschool children; employment status; job type; managerial position; and availability of flextime. WSC was assessed by using the Japanese version of an eight-item scale, and depressive symptoms, by using the Japanese version of the nine-item Patient Health Questionnaire (PHQ-9).

The Japanese version of the questionnaire developed by Kouvonen et al. was adapted [39]. Its reliability and validity have been verified [40]. This scale consists of eight items, comprising three items for bonding, two for bridging, and three for linking dimensions. Bonding items ask about willingness to work together, information sharing within the department, and mutual understanding and respect, reflecting workplace morale and colleague relationships. Bridging items ask about members generating and utilizing ideas within the department and collaborating to develop ideas. Linking items ask about the trustworthiness of supervisors and their understanding of employee rights. Responses were obtained by using the five-point Likert scale (1, strongly disagree; 5, strongly agree). In this study, the sum of the scores for the eight items was used as the WSC score, ranging from 8 to 40, with higher scores indicating higher WSC. The reliability and validity of the Japanese version have been reported as statistically acceptable [41]. 

The PHQ-9 is used as an assessment scale for depression based on the Diagnostic and Statistical Manual of Mental Disorders, Fifth Edition (DSM-5) criteria. The Japanese version was developed by Muramatsu et al. using a back-translation method with Spitzer et al., and its validity has also been examined [42]. This study used the PHQ-9 self-administered questionnaire, which consists of nine items extracted from the major depressive disorder module of the full PHQ. Symptom severity was scored as follows: not at all = 0 points; several days = 1 point; more than half of the days = 2 points; and nearly every day = 3 points, to yield a total score of 27. Scores are typically interpreted as: 0-4 = none; 5-9 = mild; 10-14 = moderate; 15-19 = moderately severe; and 20-27 = severe. While a cutoff of 10 is often used for clinical diagnosis [43], this study aimed to identify individuals who may benefit from preventive interventions rather than to make a clinical diagnosis. Therefore, we adopted a cutoff score of 5, defining scores ≥5 as "having depressive symptoms." This lower threshold is considered appropriate for screening purposes to detect even mild depressive symptoms and has been suggested as a valid cutoff in previous studies targeting general or working populations [44,45].

Statistical analysis

Logistic regression analysis was performed with the PHQ-9 score as the primary outcome. The explanatory variables were the scores for the bonding, bridging, and linking dimensions of WSC. The analysis included the adjustment variables: biological sex, age, marital status, presence/absence of children, presence/absence of caregiving responsibilities, employment status, job type, managerial position status, and availability of flextime. The PHQ-9 scores were dichotomized using a cutoff of 4 points, classifying ≤4 as "no depressive symptoms" and ≥5 as "having depressive symptoms." Biological sex, age, marital status, presence/absence of children, presence/absence of caregiving responsibilities, employment status, job type, managerial position status, and flextime availability were treated as nominal variables and analyzed by use of dummy variables. WSC scores were treated as continuous variables. Odds ratios (ORs) for having depressive symptoms (PHQ-9 ≥5) were calculated using logistic regression analysis. The logistic regression analysis involved constructing the following three models sequentially:

Model 1 (Unadjusted)

This model assessed the crude association between WSC and PHQ-9 without considering other factors.

Model 2 (Adjusted for Personal Attributes)

This model added personal attributes (biological sex, age, marital status, caregiving presence, child presence) as adjustment variables to Model 1.

Model 3 (Adjusted for Personal Attributes + Occupational Characteristics)

This model further added occupational characteristics (employment status, job type, managerial position status, flextime availability) as variables to Model 2.

By building these models step-by-step, we could examine the association between WSC and depressive symptoms in more detail while considering the influence of confounding factors. This approach allowed for a detailed investigation of the extent to which the association between WSC and depressive symptoms might be linked to other factors such as personal attributes and occupational characteristics.

Statistical analysis was performed using IBM SPSS Statistics for Windows, Version 29 (Released 2022; IBM Corp., Armonk, New York, United States), with the significance level set at 5%. The variance inflation factor (VIF) was calculated to assess multicollinearity among variables. A VIF value of <10 was considered acceptable, while a value of ≥10 was judged as potentially indicating multicollinearity.

Ethical considerations

At the beginning of the web-based survey, participants were informed about the purpose of the study, assurance of anonymity, and publication of the results. This study was conducted with the approval of the Ethics Committee of the Faculty of Medicine, University of Tsukuba (approval no. 1669-5). Protection of respondent anonymity and personal information was prioritized: the response data were anonymized, and personally identifiable information was managed appropriately.

## Results

Basic participant attributes

The survey was conducted among 21,875 individuals, and responses were obtained from 3861 of these (response rate: 17.7%). After excluding responses with incomplete data for the variables used in this analysis, the final sample consisted of 3145 valid respondents, whose basic attributes are shown in Table 1. There were 1843 men (58.6%) and 1276 women (40.6%). The largest age group was the 50-59 age group, comprising 946 individuals (30.1%).

**Table 1:** Participant characteristics

Characteristic	n	%
Sex		
Male	1,843	58.6
Female	1,276	40.6
Other	26	0.8
Age (years)		
20-29	321	10.2
30-39	583	18.5
40-49	828	26.3
50-59	946	30.1
≧60	424	13.5
Other	43	1.4
Marital status		
Single	726	23.1
Married/partnered	2,291	73.1
Widowed/divorced	119	3.8
Other	9	0.3
Caregiving		
No	2,870	91.3
Yes	267	8.5
Other	18	0.6
Childcare responsibilities		
No	1,361	43.3
Yes	504	16.0
Other	1,280	40.7
Employment type		
Regular employee	1,874	59.6
Nonregular employee	1,027	32.7
Other	244	7.8
Occupation		
Researcher	1,176	37.4
Clerical worker	972	30.9
Technician	306	9.7
Other professional/technical	245	7.8
Other	207	6.6
Missing	955	30.4
Managerial position		
Yes	476	15.1
No	2,411	76.7
Other	20	0.6
Missing	238	7.6
Able to use flextime		
No	1,376	43.8
Yes	1,529	48.6
Missing	240	7.6

Comparison of PHQ-9 in terms of personal attributes and occupational characteristics

Chi-square tests were performed to examine the presence of depressive symptoms on the basis of the PHQ-9 scores across different personal attributes and occupational characteristics (Table 2). PHQ-9 scores of ≥5 were classified as "having depressive symptoms." The group "without depressive symptoms" included 1466 individuals (52.8%), and the group "with depressive symptoms" included 1313 individuals (47.2%).

**Table 2:** PHQ-9 scores by personal attributes and occupational characteristics χ² test; PHQ-9: Patient Health Questionnaire-9 (a) indicates an adjusted residual > 1.96 (p < 0.05), and (b) indicates an adjusted residual < −1.96 (p < 0.05)

	Depressive symptoms absent		Depressive symptoms present		
	N	%	N	％	p
Sex					<0.001
Man	945	(64.5)	691	(52.8)	
Woman	512	(35.0)	602	(46.0)	
Age (years)					0.0<01
20-29	115	(7.9)^b)^	152	(11.7)^a)^	
30-39	233	(16.0)^b)^	278	(21.5)^a)^	
40-49	396	(27.3)	342	(26.4)	
50-59	444	(30.6)	403	(31.1)	
≧60	264	(18.2)^a)^	121	(9.3)^b)^	
Marital status					<0.001
Single	252	(17.2)^b)^	377	(28.8)^a)^	
Married/partnered	1,170	(79.9)^a)^	870	(66.4)^b)^	
Widowed/divorced	42	(2.9)^b)^	63	(4.8)^a)^	
Caregiving					0.03
No	1,355	(92.6)	1,185	(90.3)	
Yes	109	(7.4)	128	(9.7)	
Childcare responsibilities					0.62
No	706	(73.8)	518	(72.8)	
Yes	250	(26.2)	194	(27.2)	
Employment type					0.087
Regular employee	938	(64.1)	858	(65.4)	
Nonregular employee	521	(35.6)	453	(34.6)	
Occupation					<0.001
Researcher	679	(46.4)^a)^	458	(34.9)^b)^	
Clerical worker	393	(26.8)^b)^	520	(39.6)^a)^	
Technician	160	(10.9)	134	(10.2)	
Other professional/technical	127	(8.7)	114	(8.7)	
Other	105	(7.2)	86	(6.6)	
Managerial position					<0.001
Yes	291	(19.9)	173	(13.2)	
No	1,165	(79.6)	1,130	(86.1)	
Able to use flextime					<0.001
No	623	(42.6)	683	(52.2)	
Yes	840	(57.4)	626	(47.8)	

Regarding the association between biological sex and depressive symptoms, the proportion of "having depressive symptoms" was significantly higher among the women (46.0%) than among the men (42.2%) (p < 0.001). When the presence of depressive symptoms was compared by age group, the proportion of "having depressive symptoms" was significantly higher in the 20-29 age group (11.7%, adjusted residual +3.4) and the 30-39 age group (21.5%, adjusted residual +3.6). Conversely, in the ≥60 age group, the proportion was significantly lower (9.3%, residual -6.7) (χ² = 59.476, df = 4, p < 0.001). No clear distribution bias was observed in the 40-49 and 50-59 age groups.

Regarding the association between marital status and depressive symptoms, the unmarried group had a significantly higher proportion of "having depressive symptoms" (28.8%, adjusted residual +7.3) and a significantly lower proportion of "no depressive symptoms" (17.2%, adjusted residual -7.3). The group with a spouse or partner had a significantly lower proportion of "having depressive symptoms" (66.4%, adjusted residual -8.1) and a significantly higher proportion of "no depressive symptoms" (79.9%, adjusted residual +8.1). The widowed/divorced group showed a significantly higher proportion of "having depressive symptoms" (4.8%, adjusted residual +2.7) and a significantly lower proportion of "no depressive symptoms" (2.9%, adjusted residual -2.7) (χ² = 64.809, df = 2, p < 0.001).

When the presence of depressive symptoms was compared by job type, administrative workers had a significantly higher proportion of "having depressive symptoms" (39.6%, adjusted residual +7.2) and a significantly lower proportion of "no depressive symptoms" (26.8%, adjusted residual -7.2). By contrast, researchers had a significantly lower proportion of "having depressive symptoms" (34.9%, adjusted residual -6.1) and a significantly higher proportion of "no depressive symptoms" (46.4%, adjusted residual +6.1). No significant distribution bias was observed among technicians, other professional/technical occupations (excluding researchers/technicians, e.g., education, medical, legal, management), or other job types (χ² = 57.362, df = 4, p < 0.001).

Regarding the association between managerial position and depressive symptoms, nonmanagers had a significantly higher proportion of "having depressive symptoms" (86.1%) than managers (13.2%) (p < 0.001). Regarding the association between flextime availability and depressive symptoms, the group unable to choose flextime had a significantly higher proportion of "having depressive symptoms" (52.2%) than the group able to choose (47.8%) (p < 0.001).

Association between WSC and PHQ-9

Logistic regression analysis was conducted to examine the association between WSC and workers' depressive symptoms (PHQ-9) (Table 3). The analysis evaluated the three models with incrementally added adjustment variables.

**Table 3:** Association between WSC and PHQ-9: logistic regression analysis OR: odds ratio; CI: confidence interval; WSC: workplace social capital; PHQ-9: Patient Health Questionnaire-9 WSC was originally measured as a continuous scale (bonding: 3–15 points; bridging: 2–10 points; linking: 3–15 points). PHQ-9 low group = 0 (score ≤4); PHQ-9 high group = 1 (score ≥5). Values in bold indicate significance (p < 0.05). Model 1: Unadjusted. Model 2: Adjusted for sex, age, marital status, caregiving, and childcare responsibilities. Model 3: Additionally adjusted for employment type, occupation, managerial position, and availability of a flextime system

	Model 1			Model 2			Model 3		
WSC	OR	(95% Cl)	p	OR	(95% Cl)	p	OR	(95% Cl)	p
Bonding	0.89	0.82-0.97	0.01	0.91	0.83-0.99	0.02	0.89	0.81-0.97	0.01
Bridging	0.89	0.81-0.98	0.02	0.88	0.80-0.97	0.01	0.90	0.81-0.99	0.03
Linking	0.96	0.90-1.02	0.15	0.95	0.89-1.01	0.07	0.95	0.89-1.01	0.11
Sex									
Male				1.00 (reference)			1.00 (reference)		
Female				1.65	1.33-2.05	<.001	1.34	1.03-1.75	0.03
Age (years)									
20-29				1.00 (reference)			1.00 (reference)		
30-39				1.05	0.24-4.59	0.95	1.20	0.26-5.48	0.81
40-49				0.69	0.16-2.98	0.61	0.79	0.17-3.58	0.76
50-59				0.62	0.14-2.73	0.53	0.69	0.15-3.19	0.64
≧60				0.34	0.08-1.53	0.16	0.35	0.07-1.65	0.18
Marital status									
Single				1.00 (reference)			1.00 (reference)		
Married/partnered				0.20	0.04-1.06	0.06	0.19	0.04-1.00	0.05
Widowed/divorced				0.27	0.05-1.50	0.14	0.24	0.04-1.38	0.11
Caregiving									
No				1.00 (reference)			1.00 (reference)		
Yes				1.57	1.09-2.26	0.02	1.57	1.09-2.27	0.02
Childcare responsibilities									
No				1.00 (reference)			1.00 (reference)		
Yes				0.83	0.61-1.13	0.24	0.85	0.63-1.17	0.32
Employment type									
Regular employee							1.00 (reference)		
Nonregular employee							0.89	0.66-1.20	0.44
Occupation									
Researcher							1.00 (reference)		
Clerical worker							1.63	1.22-2.16	<.001
Technician							1.20	0.81-1.77	0.37
Other professional/technical							1.12	0.76-1.67	0.57
Other							1.25	0.79-1.97	0.34
Managerial position									
Yes							1.00 (reference)		
No							0.94	0.70-1.25	0.67
Able to use flextime									
No							1.00 (reference)		
Yes							0.77	0.60-1.01	0.06
Nagelkerke R2	0.074			0.130			0.148		

Model 1 was unadjusted. Model 2 was adjusted for biological sex, age, marital status, presence/absence of caregiving, and presence/absence of preschool children. Model 3 was further adjusted for employment status, job type, managerial position status, and flextime availability. WSC scores were treated as continuous variables in the regression. (Note: The original text mentioned analyzing WSC by comparing high and low groups, but the results show ORs are likely for continuous scores. The translation reflects the ORs provided.)

In Model 1 (unadjusted), the OR for having depressive symptoms (vs. not having) per unit increase in WSC score was 0.89 for bonding WSC (95% CI: 0.82-0.97; p = 0.01), 0.89 for bridging WSC (95% CI: 0.81-0.98; p = 0.02), and 0.96 for linking WSC (95% CI: 0.90-1.02, p = 0.15).

In Model 2 (adjusted for personal attributes), the ORs were 0.91 for bonding WSC (95% CI: 0.83-0.99; p = 0.02), 0.88 for bridging WSC (95% CI: 0.80-0.97; p = 0.01), and 0.95 for linking WSC (95% CI: 0.89-1.01; p = 0.07). Similarly to Model 1, the associations for bonding and bridging WSC were significant. Among the personal attributes included as adjustment variables, being female was significantly associated with higher odds of depressive symptoms (OR = 1.65; 95% CI: 1.33-2.05; p < 0.001). When compared with those not providing care, those providing care had significantly higher odds (OR = 1.57; 95% CI: 1.09-2.26; p = 0.02).

In Model 3 (adjusted for personal attributes + occupational characteristics), the OR was 0.89 for bonding WSC (95% CI: 0.81-0.97; p = 0.01) and 0.90 for bridging WSC (95% CI: 0.81-0.99; p = 0.03), both significant; however, no significant association was observed for linking WSC (OR = 0.95; 95% CI: 0.89-1.01; p = 0.11).

Among the personal attributes in Model 3, being female remained significantly associated with higher odds (OR = 1.34; 95% CI: 1.03-1.75; p = 0.03). Providing care also remained significantly associated with higher odds (OR = 1.57; 95% CI: 1.09-2.27; p = 0.02). Among the occupational characteristics, when compared with researchers (reference group), administrative workers had significantly higher odds of depressive symptoms (OR = 1.63; 95% CI: 1.22-2.16; p < 0.001). The Nagelkerke R² for Model 3, indicating the model's explanatory power, was 0.148. In the linear regression analysis performed to assess multicollinearity for PHQ-9 predictors, VIF values ranged from 1.033 to 3.547, indicating no issues with multicollinearity.

## Discussion

This study examined the association between the three subscales of WSC (bonding, bridging, linking) and depressive symptoms among workers in research institutions, considering personal and occupational attributes. The results of the logistic regression analysis showed that even after adjustment for personal and occupational characteristics (Model 3), higher bonding and bridging WSC were significantly associated with lower odds of having depressive symptoms (bonding OR = 0.89, 95% CI: 0.81-0.97, p = 0.01; bridging OR = 0.90, 95% CI: 0.81-0.99, p = 0.03). This finding suggests a potential protective association of WSC, particularly its bonding and bridging dimensions, with workers' mental health, independent of the adjustment factors, which is consistent with the findings of previous studies highlighting the importance of WSC for mental health [40,41].

Higher bonding WSC suggests the availability of mutual trust, reciprocal relationships, and emotional support within the workplace. Such strong colleague networks might function as a buffer during stressful events, potentially mitigating the likelihood of developing depressive symptoms [46]. Higher bridging WSC might facilitate access to diverse information, perspectives, or new resources for problem-solving through connections with people from different departments or backgrounds. This could potentially enhance individual adaptability and resilience, possibly reducing the likelihood of mental health problems. On the other hand, a significant association was not observed for linking WSC (OR = 0.95; 95% CI: 0.89-1.01; p = 0.11). This finding does not negate the importance of the relationship with supervisors, but it suggests that in this study population (workers in research institutions), horizontal relationships among colleagues (bonding and bridging) might have a stronger, independent association with depressive symptoms. This could be particularly relevant in the Japanese workplace context, where strong hierarchical structures are common. While supportive leadership (a component of linking WSC) is undoubtedly beneficial, the perceived psychological distance or formality in vertical relationships might, in some instances, dilute its measurable impact on depressive symptoms compared to the more immediate and informal support derived from peer-based bonding and bridging capital. This could be particularly relevant given that our study population consisted predominantly of nonmanagerial staff. For this group, day-to-day horizontal relationships with peers (bonding and bridging) may be more immediate and influential on their mental well-being than their vertical relationships with supervisors. This demographic characteristic of the sample might partly explain the nonsignificant association observed for linking SC. For example, cultural norms emphasizing "tatemae" (public facade) in interactions with superiors might mask underlying issues or make it harder to link capital to translate directly into mental health benefits in the same way as more open peer relationships. Alternatively, the association between linking WSC and mental health might be influenced by or confounded with other unmeasured occupational characteristics (e.g., job autonomy, specific supervisor leadership styles). Further research is needed on this point.

Focusing on the adjustment factors, being female, providing care, and being an administrative worker (as compared with researchers) were associated with a higher likelihood of depressive symptoms. This aligns with existing findings indicating potential mental health effects related to sex differences [47], caregiving burden [48], and variations in stress and job control across occupations [49], supporting the validity of our analysis. The persistence of the associations for bonding and bridging WSC after adjustment for these factors underscores the unique link of these WSC components.

The Nagelkerke R² of 0.148 for Model 3 indicates that WSC, personal attributes, and occupational characteristics combined explain only a portion of the variance in depressive symptoms. This reflects the complex nature of depressive symptoms, influenced by multiple factors, suggesting that other important unmeasured factors likely play a role.

The multicollinearity assessment (VIF < 3.6) suggests minimal statistical issues. However, the possibility of conceptual overlap between WSC subscales cannot be denied, particularly as both bonding and bridging aspects may share a common foundation of good interpersonal relationships in the workplace. Further qualitative and quantitative investigation is warranted regarding the unique phenomena captured by these scales and their interplay.

The strengths of this study include its differentiation of the structural aspects of WSC (bonding, bridging, linking) and individual assessment of their association with mental health, examination of the independent association of WSC by adjustment for diverse personal and occupational attributes, and use of standardized scales with confirmed reliability and validity (PHQ-9 [41], Japanese WSC scale [40]).

At the same time, the significant limitations of this study must be carefully considered when interpreting its findings. Crucially, the cross-sectional design fundamentally prevents the determination of causality between WSC and depressive symptoms. While we observed an association where lower bonding and bridging WSC were linked to higher odds of depressive symptoms, it is impossible to ascertain the direction of this relationship on the basis of our data. It remains unclear whether low WSC contributes to the development of depressive symptoms, whether individuals already experiencing depressive symptoms perceive or report their WSC more negatively (reverse causation), or whether unmeasured confounding factors, such as specific occupational stressors (e.g., high job demands, low decision latitude) or individual personality traits, influence both WSC and depressive symptoms simultaneously. Therefore, the observed associations should be interpreted with caution, strictly as correlations, not as causal evidence. This inherent limitation regarding causal direction is a critical constraint on the interpretation of our results.

Second, the study’s low response rate of 17.7% introduces a considerable risk of selection bias and thereby constrains the generalizability of its findings. It is plausible that participants systematically differed from nonparticipants; for example, individuals experiencing greater psychological distress might have been less likely to respond, or conversely, those with a heightened interest in workplace well-being might have been overrepresented. Such a bias could skew the observed prevalence of depressive symptoms and potentially distort the true strength or nature of the associations between WSC dimensions and these symptoms within the broader target population of workers in research institutions. Although we adjusted for several known demographic and occupational variables, unmeasured differences between respondents and nonrespondents may still have confounded the results. The potential for such confounding substantially limits our ability to confidently extrapolate these findings beyond the study sample.

Third, reliance on self-report questionnaires for both WSC and PHQ-9 may have introduced common method bias. Fourth, the participants were limited to workers in specific research-related institutions, constraining the study’s generalizability to other occupations or organizations. Fifth, as mentioned above, potentially important confounding factors (especially details of occupational stress) were not measured or adjusted for.

Considering these limitations, future research should employ longitudinal designs to examine the temporal relationship and interaction between WSC and depressive symptoms, aiming to clarify causality. Including measures related to occupational stress factors such as job content, decision latitude, and job demands would allow for a more detailed examination of whether WSC functions as a stress buffer or has an independent association. Furthermore, conducting studies with diverse occupational groups and industries would enhance the generalizability of the findings. Specifically, comparative studies across different sectors within Japan and cross-cultural studies contrasting Japanese findings with those from other countries could illuminate the extent to which cultural factors shape the role and impact of WSC dimensions. Intervention studies designed to enhance specific dimensions of WSC, particularly bonding and bridging capital in settings similar to research institutions, could also provide valuable evidence for workplace mental health promotion strategies.

In conclusion, on the basis of this cross-sectional analysis, higher bonding and bridging WSC were found to be associated with a lower likelihood of depressive symptoms among workers in research institutions, even after adjustment for several personal and occupational factors. This finding highlights a potential link between strong horizontal relationships among colleagues and better mental health outcomes in this specific population. The association with linking WSC was not significant in the final model. While these findings might suggest that fostering horizontal SC could be a potentially valuable component of workplace interventions, it is crucial to reiterate that this study design does not allow for causal inference. Therefore, firm conclusions about the protective effect of WSC or the effectiveness of interventions based solely on these results cannot be drawn. Interpretation must fully consider the study’s significant limitations, particularly the inability to establish causality and the potential for selection bias due to the low response rate.

## Conclusions

This cross-sectional study among workers in research institutions found that higher bonding and bridging WSC were significantly associated with a lower likelihood of depressive symptoms, even after adjustment for personal and occupational characteristics. In contrast, linking WSC did not show a significant association in the final model, suggesting that in this population, horizontal collegial relationships may have a more prominent association with depressive symptoms.

While these findings highlight the potential importance of fostering horizontal WSC for mental health in similar workplace settings, the study's cross-sectional design precludes causal inference. Other limitations, including potential selection bias and unmeasured confounders, also warrant caution in interpretation. Despite these limitations, this study provides foundational evidence underscoring the potential role of distinct WSC dimensions, particularly bonding and bridging capital, in relation to employee mental health within the specific context of Japanese research institutions. This contribution is notable given the relative scarcity of studies that differentiate WSC dimensions in this cultural and occupational setting. Future longitudinal and intervention studies are essential to elucidate causal relationships and to confirm the effectiveness of WSC-focused workplace interventions. Such studies should also aim to incorporate a broader range of occupational settings and delve deeper into the cultural moderators that may influence the WSC-mental health nexus, thereby informing more nuanced and effective workplace mental health strategies in Japan and potentially beyond.

Finally, our assessment of mental health was limited to depressive symptoms as measured by the PHQ-9. Other important aspects of mental well-being, such as anxiety or burnout, were not included. A more comprehensive set of mental health indicators would be needed to fully understand the multifaceted impact of WSC.
